# Even High Doses of Oral Cannabidiol Do Not Cause THC-Like Effects in Humans: Comment on Merrick et al. *Cannabis and Cannabinoid Research* 2016;1(1):102–112; DOI: 10.1089/can.2015.0004

**DOI:** 10.1089/can.2016.0036

**Published:** 2017-01-01

**Authors:** Franjo Grotenhermen, Ethan Russo, Antonio Waldo Zuardi

**Affiliations:** ^1^ nova-Institut GmbH, Chemiepark Knapsack, Hürth, Germany.; ^2^ PHYTECS, Los Angeles, California.; ^3^ Medical School–University of São Paulo, Ribeirão Preto, Brazil.

**Keywords:** cannabidiol, cannabinoid, route of administration (oral vs. transdermal), medical use, safety, THC

## Abstract

This short communication examines the question whether the experimental data presented in a study by Merrick et al. are of clinical relevance. These authors found that cannabidiol (CBD), a major cannabinoid of the cannabis plant devoid of psychotropic effects and of great interest for therapeutic use in several medical conditions, may be converted in gastric fluid into the psychoactive cannabinoids delta-8-THC and delta-9-THC to a relevant degree. They concluded that “the acidic environment during normal gastrointestinal transit can expose orally CBD-treated patients to levels of THC and other psychoactive cannabinoids that may exceed the threshold for a positive physiological response.” They issued a warning concerning oral use of CBD and recommend the development of other delivery methods. However, the available clinical data do not support this conclusion and recommendation, since even high doses of oral CBD do not cause psychological, psychomotor, cognitive, or physical effects that are characteristic for THC or cannabis rich in THC. On the contrary, in the past decades and by several groups, high doses of oral CBD were consistently shown to cause opposite effects to those of THC in clinical studies. In addition, administration of CBD did not result in detectable THC blood concentrations. Thus, there is no reason to avoid oral use of CBD, which has been demonstrated to be a safe means of administration of CBD, even at very high doses.

## Introduction

Cannabidiol (CBD) is a cannabinoid of the cannabis plant devoid of intoxicating effects. It may be of therapeutic value in a large number of diseases, including epilepsy, anxiety disorders, depression, schizophrenic psychosis, inflammatory diseases, dystonia, nausea, and vomiting without causing relevant or severe side effects.^1^

No biosynthetic enzyme or pathway exists in the human body to convert CBD to THC. However, recently Merrick et al.^2^ conducted an experimental *in vitro* study, which demonstrated that CBD rapidly cyclizes to THC in an acidic environment such as in the stomach. They concluded that patients treated with oral CBD may be exposed to significant levels of THC, which may cause unwanted psychological effects, and suggest that other delivery methods such as transdermal-based applications, which decrease the potential for formation of psychoactive cannabinoids, should be explored.

### Effects of high doses of CBD in clinical studies

In clinical studies even high doses of oral CBD did not cause THC- or cannabis-like effects.^3,4^ On the contrary, CBD caused opposite effects to THC.^5,6^ THC effects are characterized by typical psychological effects, impairment of psychomotor and cognitive performance, and a range of physical effects, including increased heart rate and dry mouth. None of these effects were observed after high doses of oral CBD.

In a study with healthy volunteers, who were given 200 mg oral CBD and alcohol or CBD alone or alcohol alone, CBD alone did not produce any impairments of motor and psychomotor performance.^7^

In healthy volunteers oral CBD in a dose of 1 mg/kg body weight reduced the anxiety provoked by THC given simultaneously in a dose of 0.5 mg/kg. This blocking of THC effects “also extended to marihuana-like effects and two other subjective alterations induced by delta-9-THC.”^8^

CBD was evaluated for symptomatic efficacy and safety in 15 neuroleptic-free patients with Huntington disease.^9^ Effects after oral CBD (10 mg/kg body weight per day for 6 weeks) or placebo (sesame oil for 6 weeks) intake were evaluated weekly under a double-blind, randomized crossover design. CBD showed no significant or clinical differences compared with placebo in the cannabis side effect inventory.

Pretreatment with 600 mg oral CBD significantly reduced anxiety, cognitive impairment, and discomfort in patients with generalized social anxiety disorder, who participated in a simulation public speaking test.^10^

It is known that THC may induce psychotic states, but CBD has been shown to produce antipsychotic effects. The first case report of a young woman diagnosed with schizophrenia, who experienced severe side effects after treatment with conventional antipsychotics, who was treated with CBD, was published in 1995.^11^ She demonstrated significant improvement of symptoms with no psychoactive and other adverse effects after 4 weeks of treatment with increasing doses of CBD up to 1500 mg/day. CBD monotherapy was administered to three patients with treatment-resistant schizophrenia (initial oral dose of 40 mg, increased to 1280 mg/day) for up to 4 weeks with no side effects reported, even at the highest dose.^12^ A similar result was observed in two patients with bipolar affective disorder who received CBD (600–1200 mg/day) for up to 24 days.^13^ CBD did not significantly affect cardiac functions or caused psychoactive effects.

The efficacy and safety of CBD on Parkinson's disease patients with psychotic symptoms were studied in a 4-week open trial.^14^ A flexible oral dose of CBD, ranging from 150 to 400 mg/day in the last week, plus patients' usual treatments showed that psychotic symptoms were significantly reduced, cognitive and motor symptoms were not affected by the cannabinoid, and no serious side effects were reported.

A double-blind study with 42 patients diagnosed with schizophrenia or schizophreniform disorder conducted at the University of Cologne showed that oral CBD (800 mg daily) significantly reduced psychotic symptoms after 2–4 weeks of treatment and induced fewer side effects, such as extrapyramidal symptoms, increased prolactin levels, and weight gain, compared with amisulpride.^15^ No relevant cardiovascular or THC characteristic psychological effects were noted.

A 19-year-old female with a history of cannabis addiction received CBD 300 mg on day 1 600 mg/day divided into two doses days 2 through 10, and CBD 300 mg on day 11.^16^ During treatment with CBD, the patient did not report any marijuana withdrawal symptoms, and she did not experience anxiety or dissociative symptoms, as assessed by standardized rating scales.

In a double-blind, crossover, placebo-controlled study with 16 healthy male volunteers, which compared the effects of 10 mg oral THC and 600 mg oral CBD, there were no differences between CBD and placebo on any investigated variable.^17^ The intake of THC on the other hand was associated with anxiety, dysphoria, positive psychotic symptoms, physical and mental sedation, subjective intoxication, and an increase in heart rate.

In a study entitled “Opposite effects of delta-9-tetrahydrocannabinol and CBD on human brain function and psychopathology” with 15 healthy men with minimal early exposure to cannabis THC and CBD had opposite effects on regional brain function.^6^ Oral THC (10 mg) and oral CBD (600 mg) had opposite effects on activation in the striatum during verbal recall, in the hippocampus during the response inhibition task, in the amygdala when subjects viewed fearful faces, in the superior temporal cortex when subjects listen to speech, and in the occipital cortex during visual processing. In a second experiment, pretreatment with intravenous CBD (5 mg) prevented that acute induction of psychotic symptoms by THC (1.25 mg).

In an open-label multicenter trial, 214 patients (aged 1–30 years) with severe, intractable, childhood-onset, treatment-resistant epilepsy were given oral CBD at 2–5 mg/kg/day, uptitrated until intolerance or to a maximum dose of 25 or 50 mg/kg/day (depending on study site).^18^ 162 (76%) patients, who had at least 12 weeks of follow-up after the first dose of CBD, were included in the safety and tolerability analysis. Adverse events reported in more than 10% of patients were somnolence in 25%, decreased appetite in 19%, diarrhea in 19%, fatigue in 13%, and convulsion in 11%. Five (3%) patients discontinued treatment because of an adverse event. No THC specific side effects were noted.

In an imaging study CBD 600 mg po functionally deactivated the left insula in human volunteers versus placebo (*p*<0.01), without accompanying sedation or other psychoactive changes.^19^ Surely, something suggestive of THC effect should have been seen with such a massive dose if the bioconversion hypothesis had any rational basis.

High doses of CBD are often required to adequately control severe epilepsy, and range from 8 to 25 mg/kg/day or more depending on whether adjunctive THC is present in the preparation.^20^ The sedation observed in a minority of patients in studies of Epidiolex occurred mostly at very high doses, particularly in patients taking concomitant clobazam, with production of excessive levels of the metabolite, *N*-desmethyl clobazam. Patients responded well to reduction of clobazam dosage with no change in CBD.^18^

## Discussion

In their study, Merrick et al. noted that oral CBD showed a relatively high incidence of somnolence and fatigue in children with epilepsy.^2^ They wondered whether these effects were due to the isomerization of CBD to THC after oral intake in gastric fluid.

However, these side effects observed in pediatric subjects, who participated in clinical studies, are not characteristic for THC. On the contrary, high oral CBD doses caused opposite effects to THC or marijuana/cannabis, for example, reduced appetite, improved cognition, and antipsychotic effects, in clinical trials.

Given the data observed in the study by Merrick et al.^2^ and the observations in clinical studies presented here, there may be mainly three explanations for these divergent results.

1. In real life, CBD may not be degraded to such a degree to psychoactive cannabinoids as under experimental conditions used in the study by Merrick et al.
2. It is known that CBD antagonizes psychological and cardiovascular THC effects, so that small amounts of THC may not influence the overall effects of CBD.
3. Dissolving CBD in methanol is inappropriate for human experiments. Using it may introduce error in extrapolating Merrick et al.'s *in vitro* study to the *in vivo* situation.

Regardless of the reason for this discrepancy, the observations in clinical studies finally count as most relevant. Thus, there is no reason to believe that the possible degradation of CBD to psychoactive cannabinoids in simulated (!) gastric fluid “may affect clinical response and lead to adverse events” (p. 111).^2^ Thus, there is no need to eliminate the potential for psychotropic effects by developing different delivery methods, such as transdermal-based systems.

## Conclusion

Even if the experimental approach taken by Merrick et al. is of interest, whether this result is of clinical relevance can only be demonstrated in the clinic. We have enough data to be reassured that the acidic gastric environment during normal gastrointestinal transit DOES NOT “expose patients treated with oral CBD to levels of THC and other psychoactive cannabinoids that exceed the threshold for a physiological response” (p. 111).^2^

Bioconversion of CBD to THC, if it occurred in fact in humans, would be easily documented through increased serum levels of THC or 11-OH-THC. This has never been demonstrated. To the contrary, available evidence would support no such effect. The plasma sample of 14 patients with Huntington disease, who received CBD (10 mg/kg/day) during 6 weeks showed that no THC was detected in the plasma sample of any patients at any time during the trial.^21^ More recently, 16 health volunteers received THC (10 mg), CBD (600 mg), and placebo in separated sessions and the plasma level of THC, 11-OH-THC, and THC-COOH were elevated at 1–3 h after THC administration, but not of CBD and placebo.^17^

The overall available scientific data, and the serum level data suggests that oral administration of CBD is a safe and easy way to use CBD, even at high doses, in a therapeutic context with no indication of human bioconversion of CBD to THC.

## References

[1] Grotenhermen F, Gebhardt K, Berger M Cannabidiol [book in German]. Nachtschatten Verlag, Solothurn, Switzerland, 2015 (1st edition), 2016 (2nd revised edition)

[2] Merrick J, Lane B, Sebree T, et al. Identification of psychoactive degradants of cannabidiol in simulated gastric and physiological fluid. Cannabis Cannabinoid Res. 2016;1:102–112 28861485 10.1089/can.2015.0004 PMC5576596

[3] Russo E, Guy GW A tale of two cannabinoids: the therapeutic rationale for combining tetrahydrocannabinol and cannabidiol. Med Hypotheses. 2006;66:234–246 16209908 10.1016/j.mehy.2005.08.026

[4] Devinsky O, Marsh E, Friedman D, et al. Cannabidiol in patients with treatment-resistant epilepsy: an open-label interventional trial. Lancet Neurol. 2016;15:270–278 26724101 10.1016/S1474-4422(15)00379-8

[5] Nicholson AN, Turner C, Stone BM, et al. Effect of Delta-9-tetrahydrocannabinol and cannabidiol on nocturnal sleep and early-morning behavior in young adults. J Clin Psychopharmacol. 2004;24:305–313 15118485 10.1097/01.jcp.0000125688.05091.8f

[6] Bhattacharyya S, Morrison PD, Fusar-Poli P, et al. Opposite effects of delta-9-tetrahydrocannabinol and cannabidiol on human brain function and psychopathology. Neuropsychopharmacology. 2010;35:764–774 19924114 10.1038/npp.2009.184 PMC3055598

[7] Consroe P, Carlini EA, Zwicker AP, et al. Interaction of cannabidiol and alcohol in humans. Psychopharmacology (Berl). 1979;66:45–50 120541 10.1007/BF00431988

[8] Zuardi AW, Shirakawa I, Finkelfarb E, et al. Action of cannabidiol on the anxiety and other effects produced by delta 9-THC in normal subjects. Psychopharmacology (Berl). 1982;76:245–250 6285406 10.1007/BF00432554

[9] Consroe P, Laguna J, Allender J, et al. Controlled clinical trial of cannabidiol in Huntington's disease. Pharmacol Biochem Behav. 1991;40:701–708 1839644 10.1016/0091-3057(91)90386-g

[10] Bergamaschi MM, Queiroz RH, Chagas MH, et al. Cannabidiol reduces the anxiety induced by simulated public speaking in treatment-naïve social phobia patients. Neuropsychopharmacology. 2011;36:1219–1226 21307846 10.1038/npp.2011.6 PMC3079847

[11] Zuardi AW, Morais SL, Guimarães FS, et al. Antipsychotic effect of cannabidiol. J Clin Psychiatry. 1995;56:485–486 7559378

[12] Zuardi AW, Hallak JE, Dursun SM, et al. Cannabidiol monotherapy for treatment-resistant schizophrenia. J Psychopharmacol. 2006;20:683–686 16401651 10.1177/0269881106060967

[13] Zuardi A, Crippa J, Dursun S, et al. Cannabidiol was ineffective for manic episode of bipolar affective disorder. J Psychopharmacol. 2010;24:135–137 18801823 10.1177/0269881108096521

[14] Zuardi AW, Crippa JA, Hallak JE, et al. Cannabidiol for the treatment of psychosis in Parkinson's disease. J Psychopharmacol. 2009;23:979–983 18801821 10.1177/0269881108096519

[15] Leweke FM, Piomelli D, Pahlisch F, et al. Cannabidiol enhances anandamide signaling and alleviates psychotic symptoms of schizophrenia. Transl Psychiatry 2012;2:e9 4 10.1038/tp.2012.15 PMC3316151 22832859

[16] Crippa JA, Zuardi AW, Hallak JE [Therapeutical use of the cannabinoids in psychiatry]. Rev Bras Psiquiatr. 2010;32 Suppl 1:S56–S66 20512271

[17] Martin-Santos R, Crippa JA, Batalla A, et al. Acute effects of a single, oral dose of delta-9-tetrahydrocannabinol (THC) and cannabidiol (CBD) administration in healthy volunteers. Curr Pharm Des. 2012;18:4966–4979 22716148 10.2174/138161212802884780

[18] Devinsky O, Marsh E, Friedman D, et al. Cannabidiol in patients with treatment-resistant epilepsy: an open-label interventional trial. Lancet Neurol. 2016;15:270–278 26724101 10.1016/S1474-4422(15)00379-8

[19] Borgwardt SJ, Allen P, Bhattacharyya S, et al. Neural basis of Delta-9- tetrahydrocannabinol and cannabidiol: effects during response inhibition. Biol Psychiatry. 2008;64:966–973 18589404 10.1016/j.biopsych.2008.05.011

[20] Russo EB Cannabis and Epilepsy: an ancient treatment returns to the fore. Epilepsy Behav. [Epub ahead of print]; DOI: 10.1016/j.yebeh.2016.09.040 27989385

[21] Consroe P, Kennedy K, Schram K Assay of plasma cannabidiol by capillary gas chromatography/ion trap mass spectroscopy following high-dose repeated daily oral administration in humans. Pharmacol Biochem Behav. 1991;40:517–522 1666917 10.1016/0091-3057(91)90357-8

